# Human Immunodeficiency Virus and Leishmaniasis

**DOI:** 10.4103/0974-777X.68528

**Published:** 2010

**Authors:** Navid Ezra, Maria Teresa Ochoa, Noah Craft

**Affiliations:** 1 *Department of Medicine, Division of Dermatology, David Geffen School of Medicine at UCLA, Los Angeles, USA*; 2 *Department of Medicine, Divisions of Dermatology and Infectious Diseases, Harbor-UCLA Medical Center and Los Angeles Biomedical Research Institute, Torrance, CA, USA*

**Keywords:** AIDS, Co-infection, HIV, Immunology, Leishmaniasis

## Abstract

The Leishmaniases are a group of diseases transmitted to humans by the bite of a sandfly, caused by protozoan parasites of the genus *Leishmania*. Various *Leishmania* species infect humans, producing a spectrum of clinical manifestations. It is estimated that 350 million people are at risk, with a global yearly incidence of 1-1.5 million for cutaneous and 500,000 for visceral Leishmaniasis (VL). VL is a major cause of morbidity and mortality in East Africa, Brazil and the Indian subcontinent. Co-infection with human immunodeficiency virus (HIV) alters the immune response to the disease. Here we review the immune response to *Leishmania* in the setting of HIV co-infection. Improved understanding of the immunology involved in co-infections may help in designing prophylactic and therapeutic strategies against Leishmaniasis.

## INTRODUCTION

The protozoan parasite *Leishmania* is highly prevalent in many areas of the world. Moreover, leishmaniasis is now gaining higher clinical importance in individuals infected with HIV-1 (human immunodeficiency virus type 1) because the distribution of both infectious agents overlaps in numerous parts of the world (e.g., Mediterranean basin, South America, India and many African countries). There is no doubt that the actual number of documented cases of co-infection is underestimated due to the various problems in recognition, diagnosis and reporting of either HIV-1 infection, or leishmaniasis or both, in the setting of developing countries. The fact that leishmaniasis is not included among the acquired immune deficiency syndrome (AIDS)-defining diseases contributes to this scarcity of information.

Leishmaniasis is transmitted to humans by the bite of a sandfly, and disease progression can be influenced by host immunity. Upon entering the host, the primary target cells are macrophages of the skin or viscera depending on the species of *Leishmania*. Immunologic control of *Leishmania* infection depends on both the innate and adaptive immune systems. About 1.5 million new cases of leishmaniasis are documented each year, and over 350 million people live in areas of active parasite transmission.[1] Co-infection of *Leishmania* and HIV has been reported frequently, yet the immunological relevance of co-infection is not well documented. Both infections affect human populations across southern Europe, Africa, the Middle East, Central and South America, Southeast Asia, and the Indian subcontinent. In this article, we compare the immunology of leishmaniasis with that of HIV and discuss the potential clinical importance of co-infection.

## HIV IMMUNOLOGY

**Figure 1:**
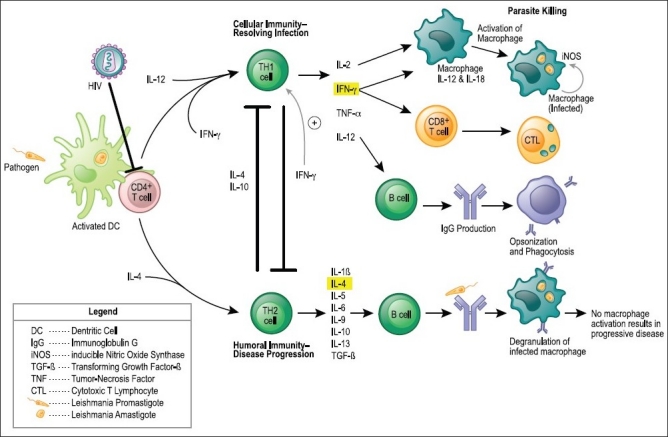
The immunology of resolving infection and disease progression to *Leishmania* For both T helper 1 (T_H_1)- and T_H_2-cell differentiations, antigens are presented to naive CD4+ T cells by dendritic cells (DCs). The interaction of co-stimulatory molecules with their respective ligands, together with the local cytokine environment, promotes the differentiation of naive T cells into interferon- γ (IFN-γ)-secreting T_H_1 cells or interleukin-4 (IL-4)-secreting T_H_2 cells. In T_H_1-cell development, certain pathogens or pathogen-associated molecular patterns (PAMPs) trigger antigen-presenting cells, through toll-like receptors (TLRs), to secrete IL-12, which promotes the differentiation of naive T cells into IFN-γ-secreting TH1 cells. In TH2-cell development, the inability of antigen to activate DCs to produce IL-12 results in a default pathway of naive T-cell differentiation into IL-4-secreting T_H_2 cells

AIDS occurs in individuals whose immune system fails due to an overload of human immunodeficiency virus (HIV) and a decreased CD4+ T cell population [Figure 1]. Opportunistic infections not commonly observed in the healthy population, such as tuberculosis, fungal infections and *pneumocystis carinii* pneumonia, are common in AIDS patients.

Since AIDS was first described over 25 years ago,[2,3] scientific advances have significantly expanded our understanding of the immune system. Following the initial burst of viremia during primary infection, HIV-infected individuals mount a robust immune response that usually curtails the levels of plasma viremia, delaying the development of clinically apparent disease for a median of 10 years. This immune response contains elements of both humoral and cell-mediated immunity directed against multiple antigenic determinants of the HIV virus, as well as against viral proteins expressed on the surface of infected cells.

CD4+ T cell subsets, designated T helper (T_H_1) that produce interferon gamma (IFN-γ), interleukin (IL)-2, IL-12, IL-15 and IL-18 are responsible for protective effects against pathogens mediated primarily through cellular immunity, whereas Th2 subsets that produce IL-4, IL-5 and IL-10 result in protective effects against pathogens mediated primarily through humoral mechanisms.[4]

Numerous immune-modulating approaches have been studied in both animal models of HIV and human vaccination.[5–7] IFN-γ, IL-2, IL-12, IL-15 and IL-21 have all been employed in multiple studies of DNA vaccines targeted against HIV or simian immunodeficiency virus (SIV).[8–13] These adjuvants help establish a strong CD8+ T cell response against antigens delivered via DNA vaccination. IL-2 and IL-12 in particular create robust anti-HIV cytotoxic T lymphocyte (CTL) responses.[9,10] IL-12, both on its own and in conjunction with IL-15, induces potent type-1 responses in rhesus macaques that have led to greatly improved clinical outcomes and control of viremia.[10,11] These studies suggest that the use of IL-12 and IL-15 in DNA vaccination against HIV or SIV leads to improved generation of CTL responses.

It has been suggested that IL-15 can prime CTLs to better degranulate in response to T-cell antigen receptor (TCR) ligation,[14] and IFN-γ may have positive results in inducing CTL activity in mouse models.[15] However, adjuvants that are effective in mouse models may not necessarily show the same potency in primates.[16]

The macrophage plays a principal role in the life cycle of HIV-1 infection.[17] A delta32 mutation in the major co-receptor for macrophage tropic HIV-1 infection, CCR5, plays a protective role, which suggests the macrophage may be the initial cell type infected by HIV-1 *in vivo*.[18] Rhesus macaques infected with simian-human immunodeficiency virus 1 SHIV-1 chimera displayed macrophage reservoirs with high viral loads of SHIV-1 even after CD4+ T cell depletion.[19] Macrophages in HIV-1-infected patients on highly active antiretroviral therapy (HAART) harbor the virus during antiretroviral therapy and release HIV-1 upon withholding drug therapy.[20,21] HIV-1-infected tissue macrophages tend to be long-lived cells that increase in number during the course of HIV-1 infection and provide a location for the expanding viral load, even during the terminal stages of HIV-1 infection, when the CD4+ T cell compartment is decimated.[17]

### Host immune response to *Leishmania*

The immune response to *Leishmania* infection is cell mediated, and clinical symptoms depend on whether the host mounts a Th1 or Th2 response.[22,23] The same parasite epitope can induce either a Th1 response with resolving infection or a Th2 response with disease progression.[24] Although the primary cell type infected is the macrophage as part of innate immunity, control of the disease hinges on the adaptive immune response.

Th1 and Th2 cells can cross-regulate each other, thereby suppressing/ enhancing each other’s effects.[4] Lymphocytes from subjects who are able to limit their infection to the protozoan parasite *Leishmania* exhibit a predominant Th1 response upon stimulation with *Leishmania* antigens *in vitro*.[25,26] Patients with active VL, in contrast, display an impaired Th1 response,[26,27] whereas Th2 responses, in particular IL-10 production, have been reported to increase.[28,29] This type of response to infection often results in disseminated disease and increased mortality.

Although much of the protection against *Leishmania* is mediated by Th1 CD4+ cells,[30] *Leishmania*-reactive IFN-γ-producing CD8+ T cells have also been associated with cure of some forms of leishmaniasis.[31] In addition, mononuclear cells from healthy individuals with no previous exposure to *Leishmania* proliferate in response to *Leishmania* antigens in conjunction with high IFN-γ and IL-6 response.[32]

Natural killer (NK) cells and CD8+ T cells also play a role in effective clearance of cutaneous leishmaniasis (CL).[33] While all three cell types are capable of secreting IFN-γ, it is IL-12 and IL-18 that direct the differentiation and proliferation of uncommitted Th cells toward the Th1 subset and stimulate the release of IFN-γ by Th1 and NK cells.[34,35]

Many cytokines play a role in the cell-mediated immunity toward leishmaniasis. IL-2, IL-3 and IFN-γ are produced by CD4+ T cells in the Th1 response to promastigotes attaching to reticuloendothelial cells.[36] IL-12 and tumor necrosis factor (TNF) play an important role in the activation of macrophages,[36] a component of innate immunity. Additionally, genetic influences on the type of immune response initiated toward the *Leishmania* species have revealed genes thought to have a major role in the outcome of infections such as those coding for natural resistance-associated macrophage protein 1 (NRAMP-1), TNF or the major histocompatibility complex.[36,37] Additionally, macrophage activation via the SLC11A1 (formerly NRAMP1) gene, and polymorphisms in the gene encoding IL-4, are also associated with increased susceptibility to VL and post-kala-azar dermal leishmaniasis (PKDL) in Sudan.[38] This supports the notion of innate immunity being the key driving force toward the adaptive host immune response.

Thus, host-determined delayed-type hypersensitivity, antigen-specific T cell reactivity and cytokine secretion all combine with the specific strain and virulence of the infecting *Leishmania* species to determine the ultimate clinical presentation of the disease.

### Innate immunity

The innate immune system comprises the cells and mechanisms that defend the host in a nonspecific manner from infection by other organisms. Although skin parasite infections are mainly controlled by adaptive, as opposed to innate, immune mechanisms, the skin innate immune system functions to detect invading parasites, recruit inflammatory cells to sites of invasion and facilitate and promote the induction of adaptive immunity. Systems such as toll-like receptors (TLRs) and complement receptors (CRs) help to detect the presence of infection and to induce activation of inflammatory and antimicrobial innate immune responses. Recognition of microbial products by TLRs expressed on dendritic cells (DCs) triggers functional maturation of DCs and leads to initiation of antigen-specific adaptive immune responses.[39] Innate immunity against *Leishmania* involves recognition receptors (TLRs), cell types (myeloid dendritic cells, plasmacytoid dendritic cells), cytokines (IL-12, IFN-α/β) and signaling pathways (Tyk2 kinase) that are necessary for the initial sensing of the parasites and the subsequent development of an efficient NK cell response.[40]

TLRs mediate activation by microbial ligands, including lipoproteins, resulting in the activation of IL-12 and nitric oxide synthase.[41] Microbial lipoproteins also induce host cell apoptosis. One of the antimicrobial products that can directly destroy pathogens, nitric oxide, is induced by TLR activation.[42] In this manner, the ability of microbial lipoproteins to activate TLRs can contribute to host defense and immunopathology during infection. More than ten TLRs have been identified in humans,[43] with keratinocytes expressing TLR1 and TLR4 and DC expressing TLR1, 2, 4 and 6.[44,45]

*Leishmania mexicana* DNA contains nonmethylated CpG motifs capable of activating murine bone marrow-derived macrophages, leading to the production of pro-inflammatory cytokines such as TNF-α and IL-12, as well as the overexpression of mRNA for TLR9.[46] CpG motifs are considered pathogen-associated molecular patterns (PAMPs) due to their abundance in microbial genomes but their rarity in vertebrate genomes. The CpG PAMP is recognized by the pattern recognition receptor (PRR) Toll-Like Receptor 9 (TLR9), which is only constitutively expressed in B cells and plasmacytoid dendritic cells (pDCs) in humans and other higher primates. Although TLR9 deficiency leads to a transient increase of IL-4, IL-13 and arginase 1 mRNA and a reduced expression of iNOS at the site of infection and in the draining lymph nodes, it does not prevent the development of Th1 cells and the ultimate resolution of the infection.[47] Therefore, TLR9-signaling is essential for NK cell activation but dispensable for a protective T cell response to *L. major in vivo*.[47] The common downstream pathways of TLR lead to the induction of various genes involved in host defense, including inflammatory cytokines, chemokines, major histocompatibility complex MHC and co-stimulatory molecules.[48]

Different patterns of chemokine expression in TLR-competent and -deficient mice after infection with *L. major* were detected depending on the post-infection time and irrespective of TLR4.[49] Genetically resistant mice lacking MyD88-adapter protein, the common downstream adaptor protein responsible for TLR and IL-1 signaling, display a high susceptibility to *L. major* infection associated with a polarized Th2 response.[50] Although the exact mechanism of action for this effect in *L. major* infections is not known, the majority of genetically deficient mice are thought to have the phenotype due to defects in TLR-signaling.[51]

Both the classical and the alternative pathways of the complement system are activated in *Leishmania* infections once promastigotes penetrate the dermis and react with serum.[52] Lysis via the membrane attack complex (C5b-C9 complex) begins 60 seconds after serum contact,[52] resulting in efficient killing of more than 90% of all inoculated parasites within a few minutes. Hence there is only a slight chance for parasite survival and establishment of an infection since most inoculations of *Leishmania* parasites are aborted early on due to complement killing.[48]

One TNF superfamily member, LIGHT, is known to enhance inflammation and T cell-mediated immunity and is important for optimal IL-12 production by DC and for the development of IFN-γ-producing CD4+ Th1 cells.[53] LIGHT is an acronym that stands for: is homologous to Lymphotoxins, exhibits Inducible expression, and competes with HSV Glycoprotein D for HVEM, a receptor expressed by T lymphocytes. LIGHT has also been called HVEM-L and LT-gamma. Its blockade results in increased susceptibility to *L. major*.[53]

Data from multiple studies point to NK cell effector function in leishmaniasis as being cytokine mediated rather than cytotoxicity mediated.[54–58] IL-12 and IL-18 are major regulators of innate and adaptive immune responses to *L. major* infection.[59,60] *L. major*- and *L. donovani*-susceptible mice are effectively cured by treatment with exogenous IL-12,[61,62] and the lack of NK cell-activating chemokines results in suboptimal NK cell-mediated defense.[63]

Rapid IFN-γ production during the first hours and days of *L. major* infection is crucial for survival, and NK cells are the initial source of this cytokine.[33,56,59] Furthermore, severe combined immunodeficient (SCID) mice, which lack T cells but have normal NK function, are able to contain *L. major* parasites in the draining lymph nodes, arguing for the existence of a T cell-independent mechanism to limit parasite spread. Neutralization of IFN-γ or depletion of NK cells prior to infection of SCID mice retards their ability to control parasite spread.[64]

Multiple studies suggest dendritic cells provide the principal source of IL-12 early in leishmaniasis, triggering NK cell activation.[65,66] Direct stimulation of the TLR-2 on NK cells by an *L. major* lipophosphoglycan (LPG) leads to up-regulation of TLR-2 and increased production of IFN-γ and TNF-α,[67] suggesting the existence of an additional, accessory cell-independent route of NK cell activation in leishmaniasis. It has been suggested that accessory cell-derived cytokines may be required for amplification of the direct response.[68]

### Adaptive immune response

Previous animal studies suggest that different strains of leishmaniasis induce different host immune responses, and that a Th1 response results in resolution of infection whereas a Th2 response results in development of severe disease.[22] The Th1 response produces INF-γ, which correlates with resistance; whereas the Th2 response produces IL-4 and correlates to susceptibility to infection.[69] In humans, IL-4, a component of the Th2 response, is also associated with disease development.[70,71]

It is well known that CD8+ T cells are essential in the defense against viruses, yet little is known of their participation in the host defense against parasites. Murine models of leishmaniasis suggest that CD8+ T cells participate through IFN-γ production.[72] There is also a large cytotoxic factor that plays an important role in cytotoxicity and apoptosis of autologous *Leishmania*-infected macrophages leading to cure.[72] It has been postulated that dendritic cells activate CD8+ T cells through mechanisms that include antigen cross-presentation, yet the exact mechanisms underlying CD8+ T cell activation in patients with leishmaniasis are unknown.[72]

The host-specific cell-mediated immunity (CMI) is important in *Leishmania* control, demonstrated by the unresponsiveness to *L. donovani* antigens[26] and the production of IL-10[29] in *L. donovani*-infected VL patients. Elevated levels of IL-10 in VL patient plasma significantly enhance the growth of *L. donovani* amastigotes in human macrophages, and IL-10-producing CD25(-)Foxp3(-) T cells may be important in the pathogenesis of human VL.[73]

### Co-infection

Our understanding of the epidemiological, biological and clinical interactions between HIV and tropical pathogens has lagged compared with our understanding of the interactions between HIV and pathogens that are common in the industrialized world.[74]

Cases of HIV and VL co-infection have been reported in 35 countries worldwide.[75] The highest prevalence of co-infection occurs mostly in Spain and southwestern Europe, with injectable drug users being at the highest risk of inoculating themselves intravenously with used syringes.[76]

The presence of both pathogens concomitantly in the same host cell (the macrophage) has enhanced reciprocal effects that influence the expression and multiplication of either one or both pathogens.[77] A vicious circle is established whereby the protozoan parasite *Leishmania* induces a more robust HIV-1 production and the virus mediates a greater parasitic replication. Indeed, it has been shown that HIV-1 infection increases the risk of developing VL by 100 to 2,300 times in areas of endemicity, reduces the likelihood of a therapeutic response and greatly increases the probability of relapse. Moreover, clinical studies have revealed that leishmaniasis promotes an increase in viral load and a more rapid progression to AIDS, which reduces life expectancy in HIV-1-infected patients. Thus, both pathogens exert a synergistic detrimental effect on the cellular immune response because they can establish infection in similar host immune cells.

Due to the depletion of both the cellular and humoral responses to *Leishmania* in co-infected patients,[78] there is an increased risk of disease progression of leishmaniasis after *Leishmania* infection in HIV+ individuals. In individuals with nonfunctional T-lymphocytes, such as in HIV-infected patients, *Leishmania* infection becomes increasingly problematic.[77]

It is thought that the parasite infection found concurrently with HIV induces chronic immune activation, and therefore an increased HIV load and accelerated progression towards AIDS.[77,80,81] In addition, immunological disturbances caused by HIV are particularly favorable for the uncontrolled multiplication of the parasite.

*L. infantum* amastigotes have been recently shown to enhance HIV-1 production in co-cultures of human dendritic cells and CD4 T cells by inducing secretion of IL-6 and TNF-α.[82] *L. donovani* can also up-regulate HIV-1 replication, both in monocytoid and lymphoid cells *in vitro* and in co-infected individuals. Not all species of *Leishmania* have been found in HIV-1-infected individuals. For example, in co-infected patients, VL is caused primarily by *L. infantum* and *L. donovani*. Other species of *Leishmania*, such as *L. ethiopica*, *L. braziliensis*, *L. major* and *L. tropica*, have been described as being responsible for cases of co-infection, according to the geographical area concerned. *Leishmania* can act as a powerful co-factor in the pathogenesis of HIV-1 infection. In those who are co-infected, complex mechanisms involving cytokine secretion and cellular-signaling events play pivotal roles in the *Leishmania*-mediated activation and pathogenesis of HIV-1.[83]

HIV infects CD4+ helper T cells, macrophages and dendritic cells. Upon infection, the virus uses the cell as a host in order to replicate. The host immune cell is eventually destroyed, leading to higher susceptibility of the organism to disease. In the Th1 response to leishmaniasis, promastigotes attach to reticuloendothelial cells, causing CD4+ cells to produce IL-2, IL-3 and IFN-γ. These molecules activate macrophages that phagocytose the promastigotes, leading to active leishmaniasis. leishmaniasis and HIV create a synergy for destruction of cell-mediated immunity and susceptibility toward opportunistic pathogens.

HIV-1 can suppress *Leishmania*-induced cellular proliferation *in vitro*.^77^ Furthermore, HIV does not affect the synthesis of cytokines TNF-γ and IL-6 induced by lipopolysaccharide or parasites.[77,84,85] Although the exact mechanism by which HIV may induce reduced cellular responses to *Leishmania* is unknown, the observed inhibitory effects of HIV on *Leishmania*-induced responses may have to do with decreased inductive signals for IFN-γ and/ or a decrease in cytokines of the Th2 type on *Leishmania*-reactive Th1 cells.[77]

Resolution of leishmaniasis infection depends on T-cell-dependent immunity and requires an intact CD4+ cell-mediated response of the Th1-cell-associated phenotype. This complex response is regulated by multiple inflammatory cytokines, including IL-2, IL-12, IL-15, IL-18, in addition to IFN-γ and TNF-α. The stimulation induces a potent intracellular leishmanicidal mechanism in phagocytes to kill residual surviving parasites.[86]

In most individuals that develop a clinically significant leishmaniasis infection, the Th1 cell response fails to fully develop. The Th2-associated CD4+ T cell response is triggered alongside IL-4, IL-5 and IL-10. It has been postulated that the antigen-specific immunosuppression observed in disseminated CL and the resulting clinical syndrome could partially be due to the ability of the infecting parasite to induce a predominance of IL-10 over IFN-γ.[87] This Th2 mechanism inhibits macrophage activation, thereby permitting intracellular replication of the parasite.[85] HIV plays an important role in induction of a Th2-like state, along with CD4+ T cell depletion, ultimately inhibiting the Th1-type cytokine secretion and increasing susceptibility to infection.[85]

Co-infection of HIV and *Leishmania* produces cumulative deficiency of host CMI, a key factor for primary protection against infection, recurrences or spread of parasites.[89] An HIV-infected patient with CL may display an atypical and severe clinical presentation of CL in terms of number (>200), sites and types of lesions (papulonodular).[89] Uncommon sites of infection are more frequent, such as the gastrointestinal tract or the upper respiratory tract.[36] Diffuse CL was seen in south India masquerading as lepromatous leprosy in the context of HIV infection.[90] There are even reports of HIV patients presenting with malabsorption, with workup revealing VL with skin and small-bowel infiltration.[91]

Co-infected patients tend to have false-negative results with the direct agglutination test (DAT) used to test for *Leishmania* antibodies,[92,93] an increased parasite load in blood and bone marrow, lower sensitivity of serological tests and a higher rate of treatment failure.[86,94] Typical symptoms that must be kept in mind when evaluating an HIV+ patient for VL are apyrexia, pancytopenia and hepatosplenomegaly. However, splenomegaly may be absent in HIV.[23]

Mucosal lesions of CL in an HIV patient should not be considered as mucocutaneous leishmaniasis, since parasites commonly disseminate and involve nasal and oral mucosa.[95,96] Diffuse CL in the absence of visceral involvement has been reported as a first manifestation leading to the diagnosis of HIV infection.[89]

In some developed countries in Europe and North America, VL is becoming a more common opportunistic infection in immunocompromised people.[86,97] VL infection in an HIV+ individual is associated with a high post-treatment relapse rate of 52% between 1 month and 3 years.[98] VL in HIV infection has been proposed for inclusion in the Centers for Disease Control and Prevention clinical category C for the definition of AIDS as an indicator disease.[97]

A recent study investigated the effect of HIV aspartyl peptidase inhibitors (PIs) on the *Leishmania amazonensis* proliferation, ultrastructure, interaction with macrophage cells and expression of classic peptidases which are directly involved in *Leishmania* pathogenesis.[99] They found that HIV PIs impaired parasite growth in a dose-dependent fashion, especially nelfinavir and lopinavir. Profound changes in the *Leishmania* ultrastructure were displayed by transmission electron microscopy, including cytoplasm shrinking, increase in the number of lipid inclusions and some cells presenting the nucleus closely wrapped by endoplasmic reticulum resembling an autophagic process, as well as chromatin condensation which is suggestive of apoptotic death.[99] The *in vitro* insight into the synergistic effects of classical antileishmanial compounds and HIV PIs in macrophages co-infected with *Leishmania* and HIV-1 should prove to be promising research.

There is an increased risk of treatment failure in leishmaniasis patients co-infected with HIV, independent of the antileishmanial pharmacologic agent administered.[100–102] A wide variety of treatment modalities exist for the diverse spectrum of clinical disease. Generic pentavalent antimonials have been the mainstay for therapy in endemic regions due to efficacy and cost-effectiveness, yet the growing incidence of resistance towards these therapeutics has seriously hampered their use.

Many of the drugs employed for the treatment of leishmaniasis, such as pentamidine and amphotericin, are toxic, marginally effective, given by injection and compromised by emerging drug resistance. Although treatment of HIV and leishmaniasis co-infection has not been adequately studied, pentavalent antimonials continue to be used extensively.[103]

Miltefosine is the first effective oral drug against VL, yet there is limited data on its role against the increasing problem of HIV-associated VL.[104] Miltefosine alone has failed to cure relapsing VL in HIV-infected patients;[105] however, it can be attempted in co-infected individuals for whom standard leishmaniasis treatment has failed.[100] Treatment with miltefosine is equally effective as standard sodium stibogluconate treatment in non-HIV-infected men with VL.[106] Among HIV-co-infected patients, miltefosine is safer, but less effective, than sodium stibogluconate.[106] Recently, miltefosine and sodium stibogluconate, in combination, have been used successfully for the treatment of an HIV-positive patient with VL.[107]

In a randomized trial comparing meglumine antimonate with amphotericin B in the treatment of VL in HIV-infected patients, relapse rate did not appear to be affected by the type of treatment given.[102] Meglumine antimonate and liposomal amphotericin have only been compared in smaller studies, in which minimal differences were found.[108] Liposomal amphotericin B is preferable to meglumine antimonate because of reduction in hospital stay; patient convenience may balance the cost of medication.[109]

Some have used secondary prophylaxis with antileishmanials to prevent relapses, and, although effective, there is no standardized regimen.[95,111] Highly active antiretroviral therapy (HAART) partially restores immune function but does not entirely prevent leishmaniasis relapses.[112,113] In contrast, a sharp decrease in the incidence of VL in Europe was observed following the widespread use of HAART, ^[114],[115]^ further supporting the notion that HAART helps prevent VL in individuals infected by *Leishmania*.^[116]^

In summary, HIV and *Leishmania* both modulate host immunity in ways that may affect the detection, pathogenesis and prognosis of the other infection. Because of an increased prevalence of co-infection in an era of continuously expanding global HIV infection, clinicians and scientists should be aware of the immune interactions between these two diseases. Additionally, astute providers in non-endemic countries for leishmaniasis should be aware of the risk of co-infection and address this possibility appropriately.
